# EDDSN-MRT: multiple rodent tracking based on ear detection and dual siamese network for rodent social behavior analysis

**DOI:** 10.1186/s12868-023-00787-3

**Published:** 2023-03-27

**Authors:** Bingbin Liu, Yuxuan Qian, Jianxin Wang

**Affiliations:** 1 Hunan Provincial Key Lab on Bioinformatics, School of Computer Science and Engineering, Central South University, Changsha, Hunan China; 2 Department of Orthopedics, Movement System Injury and Repair Research Center, Xiangya Hospital, Central South University, Changsha, Hunan China

**Keywords:** Multiple rodent tracking, Object detection, Dual siamese network, Deep learning, EDDSN-MRT

## Abstract

**Background:**

Rodent social behavior is a commonly used preclinical model to interrogate the mechanisms underpinning various human neurological conditions. To investigate the interplay between neural systems and social behaviors, neuroscientists need a precise quantitative measure for multi-rodent tracking and behavior assessment in laboratory settings. However, identifying individual differences across multiple rodents due to visual occlusion precludes the generation of stable individual tracks across time.

**Methods:**

To overcome the present limitations of multi-rodent tracking, we have developed an Ear Detection and Dual Siamese Network for Multiple Rodent Tracking (EDDSN-MRT). The aim of this study is to validate the EDDSN-MRT system in mice using a publicly available dataset and compare it with several current state-of-the-art methods for behavioral assessment. To demonstrate its application and effectiveness in the assessment of multi-rodent social behavior, we implemented an intermittent fasting intervention experiment on 4 groups of mice (each group is with different ages and fasting status and contains 8 individuals). We used the EDDSN-MRT system to track multiple mice simultaneously and for the identification and analysis of individual differences in rodent social behavior and compared our proposed method with Toxtrac and idtracker.ai.

**Results:**

The locomotion behavior of up to 4 mice can be tracked simultaneously using the EDDSN-MRT system. Unexpectedly, we found intermittent fasting led to a decrease in the spatial distribution of the mice, contrasting with previous findings. Furthermore, we show that the EDDSN-MRT system can be used to analyze the social behavior of multiple mice of different ages and fasting status and provide data on locomotion behavior across multiple mice simultaneously.

**Conclusions:**

Compared with several state-of-the-art methods, the EDDSN-MRT system provided better tracking performance according to Multiple Object Tracking Accuracy (MOTA) and ID Correct Rate (ICR). External experimental validation suggests that the EDDSN-MRT system has sensitivity to distinguish the behaviors of mice on different intermittent fasting regimens. The EDDSN-MRT system code is freely available here: https://github.com/fliessen/EDDSN-MRT.

**Supplementary Information:**

The online version contains supplementary material available at 10.1186/s12868-023-00787-3.

## Background

Rodents are highly social mammals and are typically group-housed. Therefore, as expected, social interaction models based on rodent tracking are valuable experimental tools for investigating the mechanisms underpinning disease states alongside genetics, epigenetics, and pharmacotherapy for assessment of risk, vulnerability and the development of improved treatment strategies [1–7]. Conventional rodent tracking paradigms are usually based on video recordings of behaving rodents captured by a single overhead optical camera. As such, the experimenter must distinguish video frames by the presence or absence of visual occlusion and then track rodents in occlusion and non-occlusion frames respectively. Therefore, the main challenge of multi-rodent tracking is how to correctly identify individual rodents after they touch, cross, or are occluded by one another (i.e., the occlusion condition).

Previous studies have addressed the occlusion interference problem with multi-rodent tracking primarily from three perspectives. First, is the use of social behavior models where physical contact between individuals is prevented, such as the three-chamber social test [8–10]. In these kinds of models, individuals are isolated either in individual cages or separated by Perspex walls, thus preventing conspecific interactions. However, because moving between areas is limited, such approaches do not permit a comprehensive investigation of the behavioral trajectories of spontaneous and freely behaving mice [16–19]. Second, is the use of bio-loggers (e.g., labels or tags) or special devices, such as radio frequency identification (RFID) or the use of multiple cameras during data recording [11–13]. With the aid of special equipment, these approaches can achieve a high level of tracking accuracy. However, attaching or implanting sensors into rodents has many disadvantages, such as the high cost of such devices and complex surgical requirements that could be considered an additional intervention. In addition, wearable devices and, in particular, implanted devices, may negatively impact their normal behavioral trajectories. For example, a transmitter implanted into the skull may necessitate long post-surgical recovery times, cause reduced range of motion, and loss of appetite leading to weight loss [20]. Intraperitoneally implanted transmitters have been reported to decrease spontaneous behaviors, such as running wheel activity [21, 22]. Wearable tags may negatively impact vision and olfaction, with unwanted effects on the behavior of conspecifics [23]. Third, is the use of end-to-end methods based on monocular videos with single-view depth estimation. This state-of-the-art method of Multiple Object Tracking (MOT) has been widely implemented in the tracking of pedestrians [14], vehicles [15], and animals in complex environments [16, 17, 20]. However, the inability to label recorded individuals, and the similarity across individuals’ appearances and a wide range of shapes, has led to undesirable methods to obtain accuracy, such as manual calibration and tracking over long time even when recordings are made in laboratory open field tests (OFT) with featureless, circular backgrounds.

To overcome the limitations of current methodologies, the aim of this study is to develop a behavioral recording system based on Ear Detection and Dual-Siamese Network for Multiple Rodent Tracking (EDDSN-MRT) in laboratory environments. We propose that the EDDSN-MRT system will address the aforementioned challenges in the tracking of occluded frames. We will validate our EDDSN-MRT system using a publicly available dataset on behaving mice and compare our results with a selection of state-of-the-art methods [24, 25] to determine whether our system can complete tracking operation in occlusion fragments and perform comparably, if not better, than those in current use. In addition, we will validate our system using an additional dataset to determine whether the EDDSN-MRT system can perform behavior analysis, including characterization of locomotion and movement phenotyping, and group-level location distribution profiling.

## Results

### Results for ear detection network training

We first evaluated our ear detection network (EDN) on Dataset A and compared these results with several state-of-the-art object detection methods (Table 1). Dataset A contained a freely behaving mouse in the open field test (OFT) which was determined to be suitable for training and testing ear detection.

**Table 1:** Comparison of performance for our ear detection network on dataset A

Benchmark	Epoch	Resolution	Our EDN(P)	Yolov5 %	Yolov3 %	Efficient Det %
*mAP* at *0.5*	50	1280*720	**97.07% (0.0918)**	96.15	93.22	95.41
*mAP* at *0.5:0.9*	50	1280*720	64.32%* (0.0018)	59.76	45.13	52.29

EDN, ear detection network; mAP, mean average precision.

*means P < 0.05

We found that our EDN increased mAPat 0.5:0.9 (mean average precision at Intersection over Union which is abbreviated as IoU, is from 0.5 to 0.9) by 12.03% compared with a well-known object detection model proposed in [27] (called “Efficient Det”) (52.29%), showing the effectiveness of data augmentation and adaptive anchor box functions. Due to the extreme size of subjects in Dataset A (about 5 to 8 pixels each), these two functions exerted a greater impact on performance in this dataset compared to the public dataset. However, the difference was smaller but significant in mAP at 0.5 (mean average precision at IoU is 0.5, 97.07% vs 95.41%, P = 0.0038), suggesting the metric index of the original network was already too high to be obviously improved.

When we compared the results of the object detection model YOLOv5 [28] and the EDN presented in this study, we found that our EDN had a relatively higher mAP at 0.5 (97.07% vs 96.15%, P = 0.0918) and significantly higher mAP at 0.5:0.9 (64.32% vs 59.76%, P = 0.0018) compared with YOLOv5. As mentioned above, the small difference on mAP at 0.5 may be due to the fact that the performance of the original framework is already very high and therefore it is hard to outperform. Compared to the second best-performing detection model (Yolov5), the EDN improved mAP at 0.5:0.9 by about 4%.It indicates that the EDN with our new designed Neck and Head modules achieves a better ear object detection performance.

### Results for multi-rodent tracking

At the core of the tracker is a biometric feature (ear) based algorithm which provides immense flexibility to track multiple mice. Examples of tracking videos obtained using our proposed methods are available to view in Supplementary Material (Additional file 1: Movie S1, Additional file 2: Movie S2).

As shown in Table 2, we evaluated the EDDSN-MRT system on Dataset B. This dataset is a public dataset containing 6 video clips [24]. We have numbered the videos B1 to B6. Both B1 and B2 videos contain 2 individuals. The total number of frames in B1 and B2 are 16000 and 36468, respectively. It shows that the missing IDs of the three methods (Toxtrac:idtracker.ai:EDDSN-MRT) are 8730:0:0 and 32441:0:0 (proportional figures on B1 and B2). But the ID drifting is 0:349:135 and 0:1101:730. Importantly, the results of idtracker.ai and EDDSN-MRT show that the number of missing IDs is zero. For the results of Toxtrac, the Drifting ID is zero. Numerically speaking, the detection performance of idtracker.ai and EDDSN-MRT should be better. However, due to the poor detection performance of Toxtrac, many IDs were lost, therefore the problem of ID drift is removed, i.e., since the ID cannot be detected, there is no tracking operation. The MOTAs (Multiple Object Tracking Accuracy) results were 72.6%: 97.8%: 99.1% and 55.4%:97.0%:98.0%. The ICRs (ID Correct Rate) were 67.1%:98.1%:99.5% and 38.4%:96.9%:99.0%. As such, regardless of whether MOTA or ICR was used as the comprehensive evaluation index, it was determined that idtracker.ai and EDDSN-MRT perform well, and EDDSN-MRT is comparatively better than all those tested (all P < 0.05). The performance of Toxtrac was far worse than EDDSN-MRT and idtracker.ai.

**Table 2:** Performance comparison of multiple rodent tracking on all frames in dataset B

		Toxtrac [25]					idtracker.ai [24]					EDDSN-MRT (Ours)					
ID	No	Miss	Switch	Drift	MOTA	ICR	Miss	Switch	Drift	MOTA	ICR	Miss		Switch	Drift	MOTA(P)	ICR(P)
B1	2	8730	1796	0	72.63%	67.11%	0	234	349	97.79%	98.18%	0		0	135	99.16% ( < 0.001)***	99.58% ( < 0.001)***
B2	2	32,441	12,522	0	55.42%	38.35%	0	1170	1101	96.95%	96.89%	0		16	730	98.00% ( < 0.001)***	98.98% ( < 0.001)***
B3	2	11,257	7195	0	72.95%	55.83%	252	3800	491	97.01%	89.12%	0		10	451	97.84% ( < 0.001)***	98.90% ( < 0.001)***
B4	2	7907	13,903	0	80.95%	47.81%	66	332	180	98.96%	98.62%	0		0	185	**99.11%(0.117)**	99.56% ( < 0.001)***
B5	4	64,370	59,250	0	68.19%	39.34%	2175	13,726	0	98.93%	92.20%	0		14	832	99.18% ( < 0.001)***	99.58% ( < 0.001)***
B6(1)^1^	4	None^2^	None	None	None	None	9040	7420	0	96.43%	89.02%	0		24	1240	98.34% ( < 0.001)***	99.16% ( < 0.001)***
B6(2)^1^	4	None^2^	None	None	None	None	6800	4120	0	96.90%	92.54%	0		0	1261	98.28% ( < 0.001)***	99.14% ( < 0.001)***

^1^Since there is an occluded fragment in video B6 caused by manual operation, individual information cannot be obtained in this fragment. Therefore, video B6 is divided into two parts to implement our method after deleting this fragment. idtracker.ai is not affected by this occlusion

^2^Toxtrac does not run on video B6, therefore no results are presented

MOTA, Multiple Object Tracking Accuracy; ICR, ID Correct Rate. No., the number of rodents in the video. ***means P < 0.001

The duration and frame numbers of videos B3 and B4 were very close, therefore they are combined for discussion. Unlike the results of B1 and B2, the number of Missing IDs was not zero for the idtrackerai’s results of B3 and B4. Therefore, these results are made with undetected IDs in both videos, and the number of errors due to ID switching increased dramatically, greatly exceeding the number of errors due to ID drifting. This also indicates that idtracker.ai has degraded performance on these two videos. Compared with the issue of a large increase in ID missing in the output of idtrakcer.ai, the number of Missing IDs in the result of EDDSN-MRT was still zero. This indicates that the performance of EDDSN has not declined while the difficulty of tracking individuals in the video increasing, demonstrating the superior performance of the EDDSN-MRT system. The performance on B3 and B4 of Toxtrac was similar to that on B1 and B2 inasmuch as a large number of Missing IDs occurred, demonstrating poor performance in object detection. The MOTA and ICR indicators of the three methods on B3 and B4 were also similar to those on B1 and B2, with EDDSN-MRT getting the highest score, idtrackerai second, and Toxtrac the worst.

Video B5 and B6 are different from the previous videos in that they contain 4 mice in each. The idtrackerai and EDDSN-MRT were run on these two videos for comparisons. Due to the occlusion caused by manual operation for a period in B6, it was cropped into two segments for the EDDSN-MRT run. Toxtrac could not be run on B6, resulting in missing data for these two videos (Table 2).

As the results in Table 2 demonstrate, our EDDSN-MRT method consistently generates output with no missing IDs, sporadic ID switching and ID drift. This suggests that the performance of our method has not degraded in this kind of video, where more subjects are present, and the video duration time is longer. By comparison, the performance of Toxtrac and idtracker.ai show greater degradation. It is worth mentioning that the number of ID drift errors in the results of idtracker.ai have been reduced to 0 at this time (similar to the results of Toxtrac). However, there are still a lot of ID drift errors in EDDSN-MRT. As in the previous analysis, the number of ID drift errors dropped to 0 does not mean better tracking performance. Rather, because the detection performance is so poor, most video frames do not even enter the stage of ID tracking. If we observe the two-evaluation metrics from a global perspective, we will find that MOTA is insensitive to ID switching errors. The fact that only a few ID switches occur but the mice hold the wrong ID for a long period of time does not significantly reduce the MOTA assessment.

We repeated the above assessment on occlusion frames within Dataset B to verify the robustness of our method where subjects are occluded. We compared the tracking performance of all three methods in occlusion frames of the videos used (Table 3). The results show that EDDSN-MRT performs significantly better than Toxtrac and idtracker.ai in terms of ICR and MOTA in occluded frames (all P < 0.001).

**Table 3:** Performance comparison of multiple rodent tracking on occluded frames in Dataset B

		Toxtrac[25]					idtracker.ai[24]						EDDSN-MRT (Ours)				
ID	No	Miss	Switch	Drift	MOTA	ICR	Miss	Switch	Drift	MOTA	ICR	Miss		Switch	Drift	MOTA(P)	ICR(P)
B1	2	6651	619	0	9.84%	1.42%	0	218	301	89.82%	90.84%	0		0	119	97.05% ( < 0.001)***	98.52% ( < 0.001)***
B2	2	20,964	1151	0	10.65%	6.1%	0	986	430	92.54%	90.29%	0		14	721	94.12% ( < 0.001)***	96.97% ( < 0.001)***
B3	2	8412	216	0	4.25%	3.91%	176	1504	443	72.27%	60.80%	0		6	451	90.19% ( < 0.001)***	95.00% ( < 0.001)***
B4	2	7737	4656	0	49.82%	30.97%	40	182	170	97.72%	97.68%	0		0	185	98.50% ( < 0.001)***	99.25% ( < 0.001)***
B5	4	61,210	15,080	0	20.26%	4.50%	1892	8112	0	89.54%	81.07%	0		2	832	98.26% ( < 0.001)***	99.13% ( < 0.001)***
B6(1)^1^	4	None^2^	None	None	None	None	3429	1622	0	79.81%	73.32%	0		12	1238	90.05% ( < 0.001)***	94.95% ( < 0.001)***
B6(2)^1^	4	None^2^	None	None	None	None	2756	2130	0	76.02%	65.57%	0		0	1211	88.11% ( < 0.001)***	94.06% ( < 0.001)***

MOTA, Multiple Object Tracking Accuracy; ICR, ID Correct Rate. No., the number of rodents in the video. ***means P < 0.001

### Ablation study for Video B1

To verify the effectiveness of each component in EDDSN-MRT, we designed an ablation study for Video B1. The first component of the ablation study was designed to demonstrate the effectiveness of ear detection-based methods (EDB) using tracking with traditional torso detection-based methods (TDB) (Table 4).

**Table 4:** Results of the ablation study to verify the effectiveness of each EDDSN-MRT component

Method	ICR	ID	^1^P value
TBD + IEDN + DSN	86.77%	16,000	P < 0.0001
EBD + EDN + DSN	98.20%	16,000	P < 0.0001
EBD + IEDN + SSN	67.40%	16,000	P < 0.0001
EBD + IEDN + DSN	**99.58%(P < 0.0001)**	16,000	–

^1^The P value is obtained by comparing the ICR of each other method and the EDB + IEDN + DSN method

EBD, Ear Based Detection method; EDN, Ear Detection Network(original); DSN,Dual-Siamese Network; ICR,ID Correct Rate; IEDN,Improved Ear Detection Network; TBD,Torso Based Detection method; SSN, Single-Siamese Network

The results indicate that TBD + IEDN + DSN used the rodent torso as the target to implement object detection (Torso Based method, TDB) and perform tracking operations, which performed well in terms of correct IDs (86.77%). However, using the ears as targets improved object detection and correct IDs (99.58%), and performed significantly better than TBD (P < 0.0001).

The second component of the ablation study was to demonstrate the effectiveness of the improved ear detection network (IEDN), which uses ear detection with the original PANet (EDN). The second and fourth row of Table 4 shows that improved PANet can significantly improve ICR from 98.2% to 99.58% (P < 0.0001). Combined with the data shown in Table 2, it is clear that the object detection framework using enhanced PANet has a greater ability to locate targets (improved mAP at 0.5:0.9 from 58.58% to 64.32%, P < 0.0001), making this method suitable for variable environments. These results also indicated that the IEDN is effective for both rodent detection and tracking.

The dual-Siamese network framework used in this study has two independent Siamese networks: one is used to process image information of rodent subjects, and the other one is used to preserve spatial information. To show the effectiveness of the dual-Siamese network, we compared its performance with the traditional Single-Siamese network (SSN), which only processes images to validate the effectiveness of DSN. The ICR of DSN is 32.17% higher than the one of SSN (Table 4, P < 0.0001). The reason may be that the area of the mouse ear is very small—even in 1920 × 1080 resolution, it is still only 30 × 30 pixels in size. Furthermore, it is difficult to solely use image features for tracking without using spatial information for constraints. These factors validate the necessity of DSN and also show how the presence or absence of spatial information can have a big impact on the performance of the entire tracking framework.

### Mouse experiment validation

#### Results of velocity

**Fig. 1:**
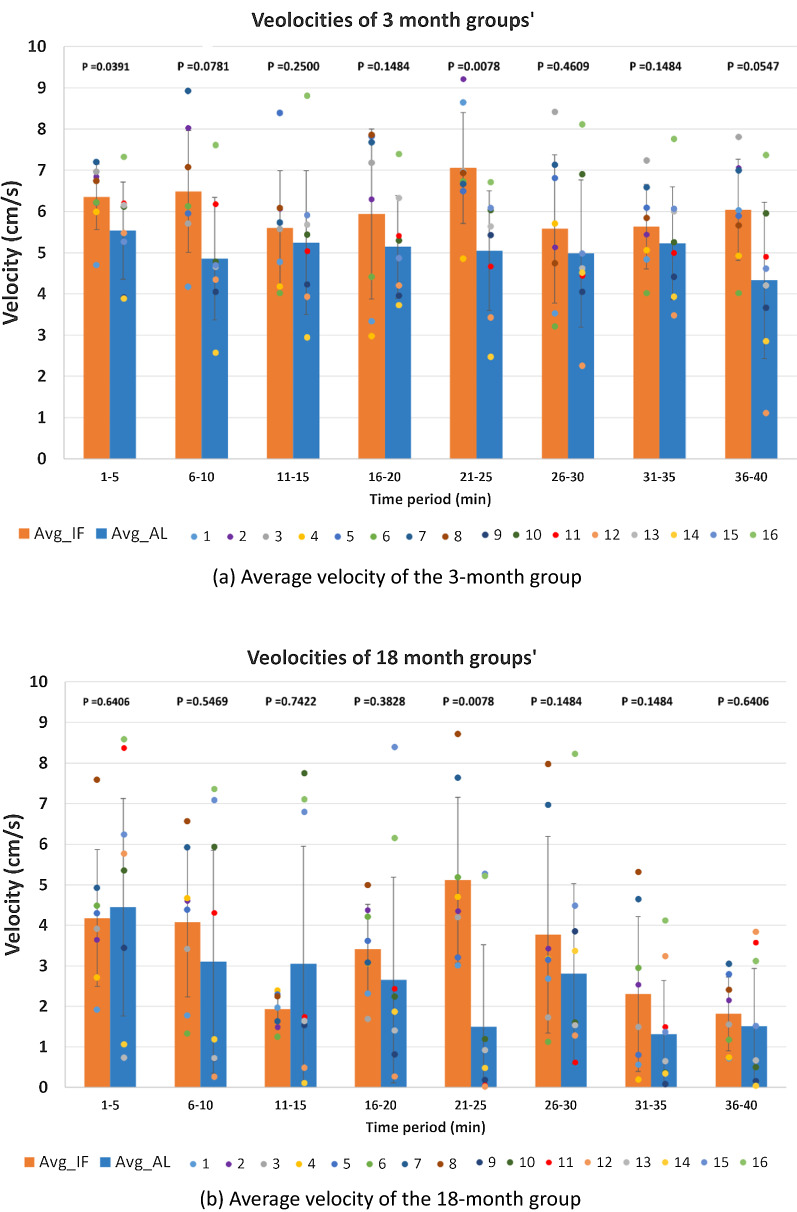
Monitoring of individual and group locomotion characteristics—Assessment of velocity. **a** Average velocity of the 3-month group (n = 8) for both intermittent fasting (orange bars) and ad libitum feeding groups (blue bars), and **b** average velocity of the 18-month group (n = 8) for both intermittent fasting (orange bars) and ad libitum feeding groups (blue bars). All data are presented in 5-min time blocks. Bars indicate group-level averages, error bars indicate standard deviation, and individual dots represent individual subjects (mice)

We monitored the movement of 32 subjects and obtained 32 tracking trajectories, the average velocities of each subject, and the velocities of each subject per 5-min time block (the video is 40 min in total). Compared with the single-session experiment, the group analysis reveals diverse locomotion characteristics. It has been suggested that as individuals age, damaged mitochondria produce less adenosine triphosphate (ATP) and more reactive oxygen species (ROS) accumulate, resulting in depression-like symptoms and in turn a weakening of locomotion ability [29, 30]. This was also observed in the results of this experiment (Fig. 1), where the older mice (aged 18 months) demonstrate a lower average velocity in both the AL (ad libitum feeding) and the IF (intermittent fasting) groups (both P < 0.05). Compared with the older mice, the younger mice (aged 3 months) with the same feeding schedule had the greater frequency of ambulation. According to the previous research [31], an IF intervention may alleviate depressive symptoms, which could improve locomotor performance and range of motion of monkeys and rodents.

We recorded the average speed of mice of each group over 40 min (2400 data per group) and performed Wilcoxon rank sum test on the speed data of two groups of mice in the same age. There were significant differences in velocity between IF and AL mice in both young and old groups (both P < 0.001). And IF mice had significantly higher average velocities compared with AL mice in young (6.08 vs. 5.04 cm/sec) and old (3.32 vs. 2.54 cm/sec) groups, consistent with previous findings [31]. In order to clarify in which time period the difference in velocity primarily occurred, we performed the Wilcoxon Rank Sum Test on both age groups within the 40-min time period in 5-min units. We found that significant differences in velocity were concentrated in the 21-25 min period (P < 0.05 in both age groups) (Table 5). This pattern was observed in both young and old age groups. Furthermore, we observed that IF mice were more active than the average level of activity during this period (Fig. 1a, b), which was not found in the AL mice.

**Table 5:** Wilcoxon Rank Sum Test results on the velocity of intermittent and ad libitum feeding mice in young and old age groups across 5-min units of time

Time (min)	1–5	6–10	11–15	16–20	21–25	26–30	31–35	36–40
3-month	0.0391	0.0781	0.2500	0.1484	0.0078	0.4609	0.1484	0.0547
18-month	0.6406	0.5469	0.7422	0.3828	0.0078	0.1484	0.1484	0.6406

#### Spatial distribution of mice and time spent in a specific location

**Fig. 2:**
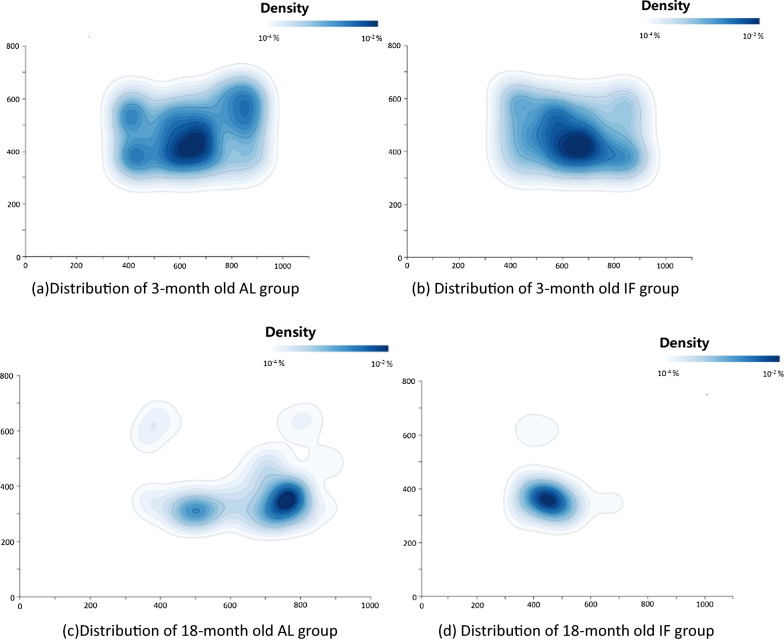
The spatial distribution of mice (n = 8 per graph) and time spent in a given region. Histograms indicating spatial location and time spent in the location for all mice in each of the feeding regimens and age groups. Graph **A** shows the distribution of the 3-month-old AL group, **B** shows the distribution of the 3-month old IF group, **C** shows the distribution of the 18 month old AL group, and **D** shows the distribution of the 18-month old IF group. Each histogram was constructed by computing the percentage of time spent in a given pixel. Data were smoothed and presented in log scale

The AL mice in the 18-month age group were walked further and were more widely distributed within their environment (Fig. 2A–D). By contrast, mice in the IF group were more likely to cluster together. This phenomenon was most observed in the older, 18-month-old mice. To quantify this, we calculated the two-dimensional (2D) standard deviation distribution coordinates of these mice. The standard deviation in 2D Euclidean space is the extension form of the one in 1D space and can be calculated as follows (Eq. 1):

1 $$\sigma = \sqrt {\frac{{\mathop \sum \nolimits_{i = 1}^{n} \left( {x_{i} - \overline{x}} \right)^{2} }}{n}}$$

However, mice are distributed within a 2D matrix with two variables, x (horizontal coordinate) and y (vertical coordinate). Therefore, to extend Eq. 1 to a 2D matrix, it is written as follows (Eq. 2):

2 $$\sigma = \sqrt {\frac{{\mathop \sum \nolimits_{i = 1}^{n} \left( {x_{i} - \overline{x}} \right)^{2} + \left( {y_{i} - \overline{y}} \right)^{2} }}{n}}$$

The result shows that in the young mice group, the standard deviation of the AL and IF mice is 20.62 cm vs. 19.05 cm, respectively. In the old mice group, the standard deviation of the AL and IF mice is 18.59 cm vs. 15.82 cm, respectively. These results demonstrate that in both age groups, the AL mice have a larger spatial distribution.

**Fig. 3:**
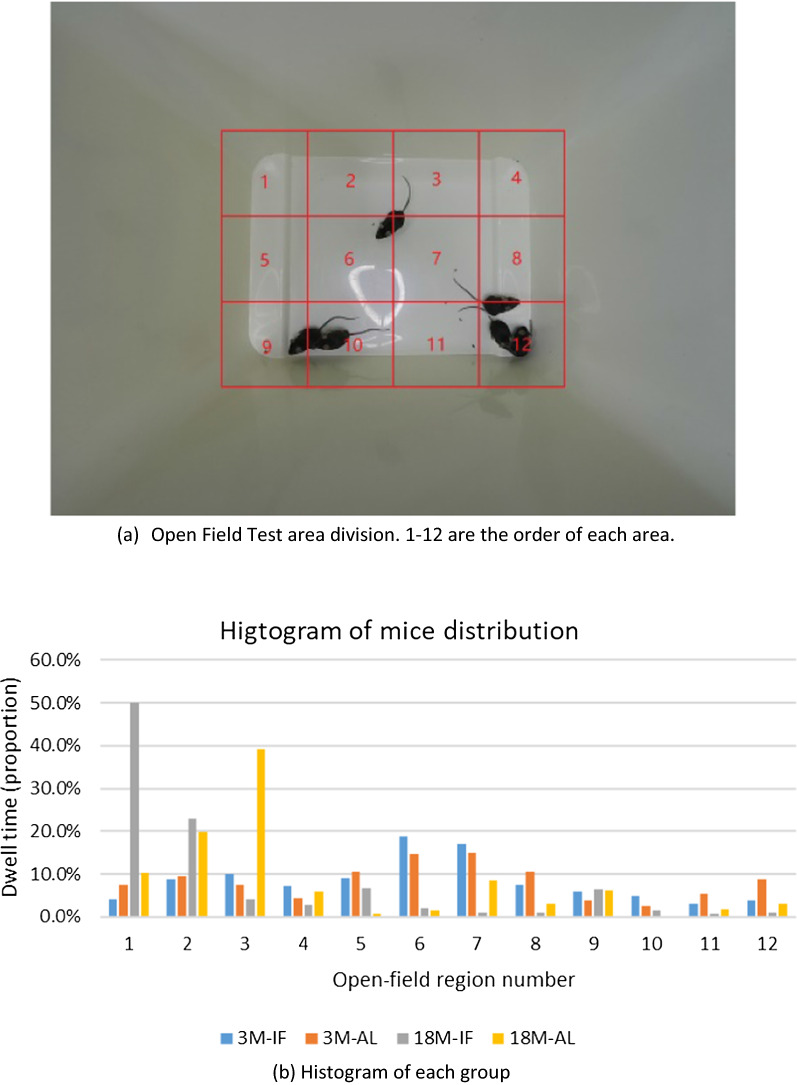
Representative photo of mice in the open field test and histograms of each group’s distribution and time spent in each location within the open field test. **A** The open field was divided into 12 regions for analysis, and **B** histograms were created to show the spatial distribution of mice and time spent in each location. OFT, open field test

**Fig. 4:**
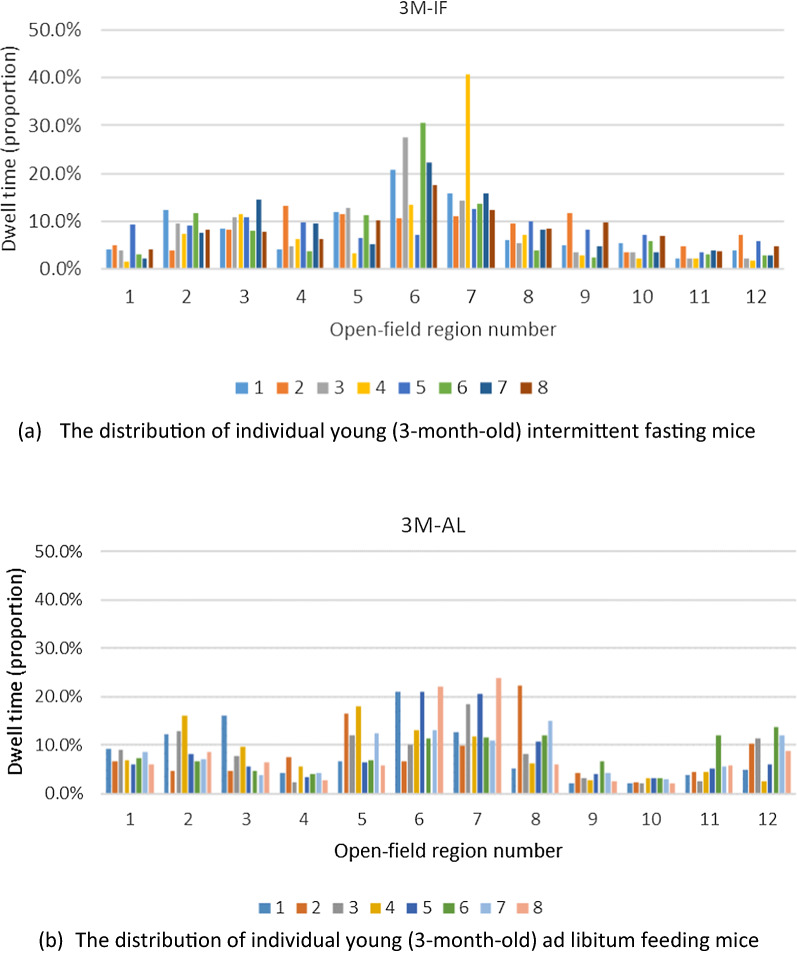

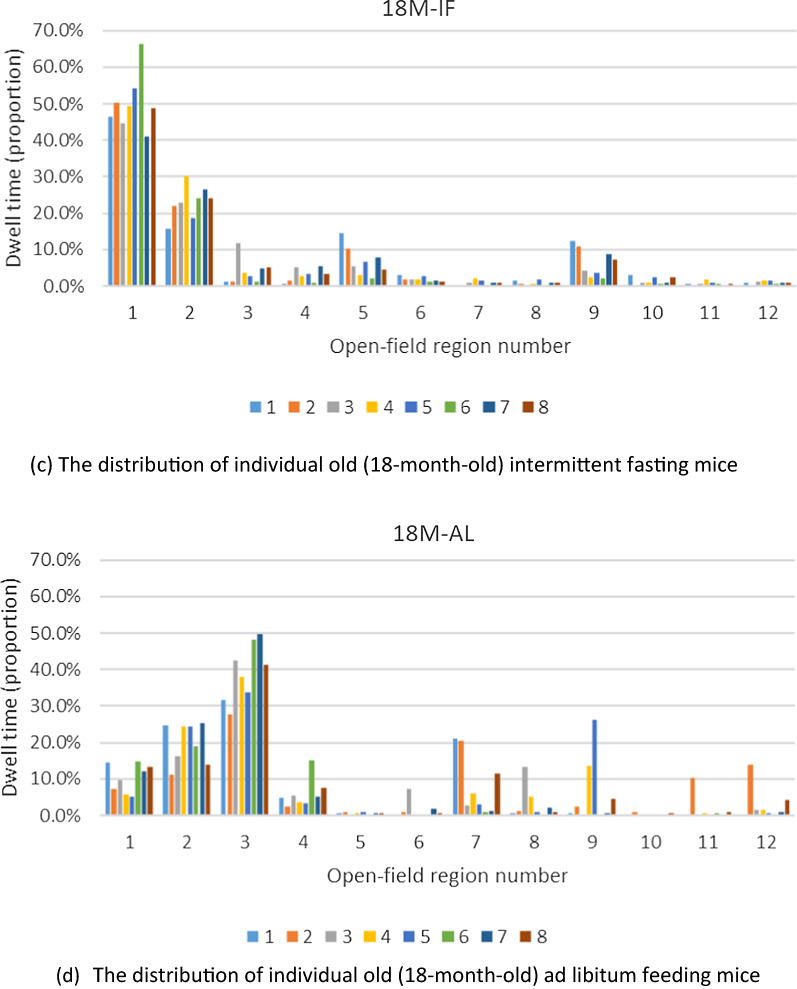
Histograms showing each individual’s spatial distribution and proportion of time (%) spent in each of the twelve locations within the open field test

In order to reduce the error caused by the difference in areas of activity in individual mice versus the overall activity area of the group, we analyzed the activity area of every mouse separately. Since the video resolution is 1280 × 960, we divided the main region of the open field (from 320 to 960 on the horizontal axis, and 240 to 720 on the vertical axis) into 12 regions. Each region was 160 × 160 in size and numbered 1–12 (Fig. 3a) and histograms were generated for all groups (i.e., young vs. old mice, and AF vs. IF mice) (Fig. 3b). Finally, we plotted the histograms for each individual mouse to represent their location within the open field test and the proportion of time each mouse spent within the twelve described locations (Fig. 4). Although significant differences were not found (Wilcoxon Rank Sum Test), a trend was observed suggesting that the IF mice preferred to stay in fewer areas compared to the AL mice, and the space within which IF mice were distributed was far smaller than the AL mice. This finding was consistent across both individuals and groups.

## Discussion

This study presents a novel approach for ear detection, the EDDSN-MRT system, which avoids occlusion interference in multiple object detection analyses. This approach makes multiple rodent tracking based on object detection accessible and is an improvement on whole-body detection which is vulnerable to occlusion. To adapt the EDDSN-MRT system for detecting ears of small sizes, we improved the existing PANet structure to obtain more detailed features from low-level layers. In the conventional architecture of object detection networks, PANet is an independent component for feature extraction. Therefore, this improvement could be applied to most current ODNs similar to Yolo. Furthermore, it is feasible that the EDDSN-MRT system would be compatible and adaptable to a new ODN with better performance in the future. Since spatial and image information is extracted by an ODN, we used a dual-Siamese-network to measure the similarity between images of a pair of ears and spatial information in adjacent frames to assign identification to individual mice.

Comprehensive and unbiased locomotion phenotyping is an emerging and powerful approach for assessing abnormal social behaviors in animal models of mood and depressive disorders [29–31]. In this study, we validated the application of the EDDSN-MRT system in the monitoring of social behavior of intermittent fasting and ad libitum feeding mice of different ages. Interestingly, we found that mice with an intermittent fasting intervention were significantly more active in spontaneous movement compared to the ad libitum feeding mice. This difference was most obvious in the 20–25-min timeframe (Table 5, both P = 0.0078). Previous studies have suggested that an intermittent fasting intervention could modulate mood and social behaviors in rodent models, relieving symptoms of depression and anxiety in mice [34–36]. This relief of symptoms would be evinced by an increase in spontaneous locomotion and a larger dwelling distribution of mice. However, the results of the open field test presented here showed the opposite findings. Compared with the ad libitum feeding mice, the mice with the IF intervention had a smaller dwelling range. This could be interpreted as a sign of stable or increasingly worse depressive and/or anxiety symptoms. However, it is well-known that fasting induces a lower body temperature [37–44]. This likely results in reduced physical agility and the desire to maintain body temperature by clustering, leading to a smaller range of locomotion. Therefore, intermittent fasting not only impacts on the mood of mice, but also on their physiological functioning.

Lastly, we would like to discuss the limitations of our proposed system. Mouse (or rodent) ears are a type of biometrical characteristic (BMC), but the BMC tracking performance heavily depends on the ODN designed for the specific feature. However, in some cases using ears for rodent tracking may be unreliable because of various problems such as not all rodents have such distinctive ears, and some types of rats (e.g., those with white fur) show very slight differences between the fur colour and the ear colour, In this case, it is difficult to identify the ears well, thus, we would need to select a new BMC for tracking. As a next step, we are considering using generative adversarial network or semantic image segmentation to generate visible BMC marks for rodent subjects to enhance the performance of the ODN. Solving these problems will extend the applicability of our framework to the benefit of the animal behavioral research community.

## Conclusion

The EDDSN-MRT is an automated pipeline framework for multiple rodent tracking. The system is robust to solve the occlusion problem in multiple individual tracking via tracking rodent ears as opposed to the entire rodent’s body. EDDSN-MRT can greatly improve the study of rodent movement and behavior by reducing the video processing time, avoiding observer bias, and allowing transparent, reproducible workflows. Experimental results show that when compared with the current approaches, our proposed EDDSN-MRT achieves better performance in identification assignment for tracking individual mice. It also helped us to observe unexplained effects of intermittent fasting on rodent behavior in the laboratory.

### Method

In the following sections, we demonstrate several advantages of the EDDSN-MRT system for tracking multiple rodents compared with several existing state-of-the-art animal tracking object detection methods using multi-rodent behavior datasets.

### Experimental procedures

**Fig. 5:**
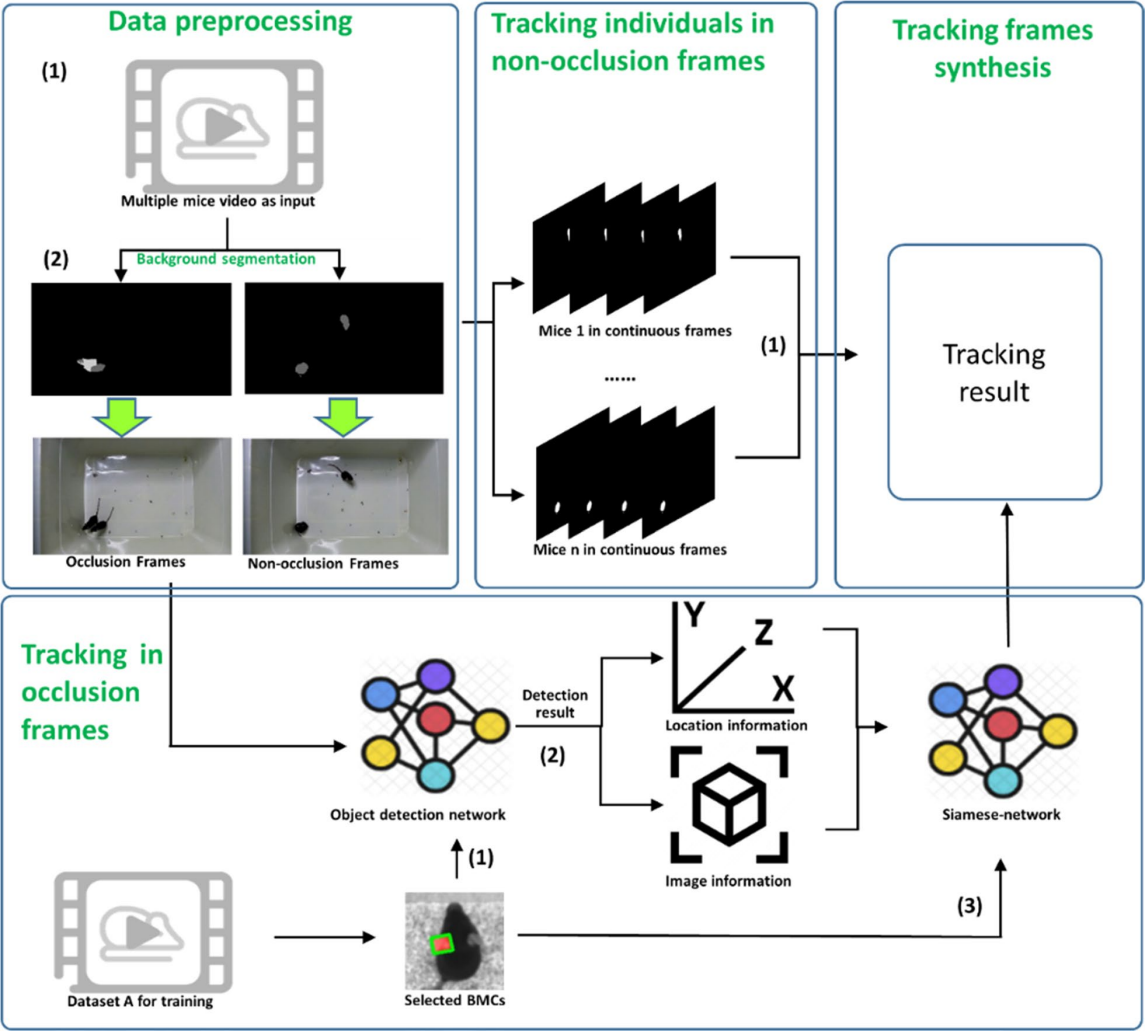
The pipeline of the proposed EDDSN-MRT system. Data preprocessing: (1) recordings are captured from a single optical camera; (2) frames with and without individual occlusion are identified; Tracking individuals in non-occlusion frames: (1) an algorithm based on blob overlapping is used to assign identities; Tracking in occlusion frames: (1) the ear detection network is trained with images of labeled ears; (2) the object detection network is used to characters the spatial and image features of individual ears; (3) a dual-Siamese network is trained using the spatial and image features of individual ears; Final Tracking Result Generation: the final result is a combination of tracking in both occlusion and non-occlusion frames

We first divided frames into occlusion frames and non-occlusion frames via a segmentation process, followed by implementation of tracking operations (see Fig. 5 for the pipeline of the proposed EDDSN-MRT system). Because of the occlusion of individuals, some blobs in occlusion fragments could contain multiple individuals in space. As such, it was not possible to assign identification directly in the same manner as that in non-occlusion frames. To overcome this, the following three steps were implemented for the tracking operation in occlusion frames. The first step was ear labeling. Before tracking in occlusion frames, we first selected rodent ears as the key points for tracking since they are least likely to be occluded by individuals touching or crossing. The ear images, as opposed to the whole body, were used as machine learning input in order to train an ear detection network (EDN) based on Path Aggregation Network (PANet) [26] to locate and identify the ears of individual rodents. This step enabled the extraction of the ears’ (and individuals’) position in space and its image characteristics. In addition, we utilized a dual-Siamese network for spatial information and image characteristics of the detected ears as additional input to calculate the similarity between two frames that were used to assign identification of each rodent. Within the EDN, similarity calculating and ID assigning in occlusion fragments were performed. We then tested the EDDSN-MRT system using a publicly available dataset on behaving mice. We compared the results of the EDDSN-MRT system with a selection of state-of-the-art methods [24, 25] to determine whether our system could complete tracking operations in occlusion fragments and perform comparably, if not better, than those in current use. In addition, we validated our system using an additional dataset to determine whether the EDDSN-MRT system could perform behavior analysis, including characterization of locomotion and movement phenotyping, and group-level location distribution profiling.

### Datasets

#### Dataset A

This unpublished dataset contains one clip which was used in training and testing of the object detection network (ODN). The video stream was recorded inside a glass chamber (size 50 length × 30 width × 35 height cm^3^). The chamber did not have a roof and the walls were high enough to prevent the mice from escaping. The bottom of the cage was covered with a polyvinyl chloride plastic sheet for building a featureless background. The camera was set 50 cm from the top of the ground. The sample was one male C57BL/6 J mouse (aged 3 months, obtained from the SLRC laboratory animal center, Shanghai, China) who was single-housed in an individually ventilated cage (Type 500) in a temperature (22° ± 2 °C), humidity (45–65%) and light controlled room with a 12–12 h light–dark cycle (12 h of lights on starting at 6:00 am, and 12 h of lights off starting at 18:00 pm). The length of the video for training and testing was 17 min and 20 s, with 51,600 frames in total. The clip had 1080P original resolution and 60FPS frame rate. Details of Dataset A are shown in Table 6.

**Table 6:** Details of the three datasets used in this study

Dataset	ID	Video name	Number of samples	Age	Intervention type	Frames per second	Duration	Resolution
A	A1	1C57BL	1	3-mouth	None	60	17′20”	1920 × 1080
B	B1	2aguties	2	Unknown	Unknown	49	05′19”	984 × 557
B	B2	2negroscanosos	2	Unknown	Unknown	49	12′24”	984 × 557
B	B3	2negroslisocanoso	2	Unknown	Unknown	49	07′06”	984 × 557
B	B4	2negroslisos	2	Unknown	Unknown	49	07′06”	984 × 557
B	B5	4 black mice (1)	4	Unknown	Unknown	25	33′58”	1272 × 909
B	B6	4 black mice (1)	4	Unknown	Unknown	24	52′54”	1272 × 894
C	C1	3 m-IF	8	3-mouth	Periodic fasting	48	40’	1280 × 720
C	C2	3 m-AL	8	3-mouth	None	48	40’	1280 × 720
C	C3	18 m-IF	8	18-mouth	Periodic fasting	48	40’	1280 × 720
C	C4	18 m- AL	8	18-mouth	None	48	40’	1280 × 720

#### Dataset B

Dataset B is a public dataset that contains 6 clips of video used for validation of tracking systems performance [24]. Two videos with four mice were recoded inside a translucent plastic cage (size 30 length × 47 width × 35 height cm^3^) chamber inside a bigger tank made of glass. There was no roof on the chamber and the walls were high enough to prevent the mice from escaping. Four videos with two mice were recorded in a transparent plastic cage (size 18 × 32 × 20 cm^3^) covered with a transparent Perspex roof to prevent the mice from escaping. In both cases, the bottom of the cage was covered with sawdust for the comfort of the animals. Cameras were set around 110 cm and 100 cm from the top of the ground for the four-mice and two-mice videos, respectively. With the exception of the agouti mouse in the video named *2aguties*, the other mice were presumed to be C57BL mice. Details of Dataset B are also shown in Table 6.

#### Dataset C

This dataset (unpublished) contains 4 clips of video used for monitoring of rodent movements in experiments. The video streams were recorded inside a plastic cage size 60 length × 45 width × 37 height cm^3^). There was no roof on the cage and the floor was uncovered. The camera was set 50 cm from the top of the floor. The sample was a group of male C57BL/6 J mice (n = 32 subjects in total) obtained from the SLRC laboratory animal center, Shanghai, China). Two groups were obtained, one aged 3-month and the other, 18-months (n = 16 for each age group). The mice were housed in IVC cages (Type 500, 4 mice per cage) in a temperature (22° ± 2 °C) and humidity (45–65%) controlled room with 12–12 h light–dark cycle (12 h of lights on starting at 6:00 am, and 12 h of lights off starting at 18:00 pm).

For each age group, the mice were divided into intermittent fasting (IF) and ad libitum feeding (AL; the sham group) groups (as shown in Table 6). The paradigm of IF involves periodic dietary restriction in a fasting week, in which the mice are fasted every other day, i.e., fasted one day and fed ad libitum one day. Feed pallets for the IF group were provided or removed at 10:00 am every day. The periodic fasting operation lasted for one week, and the mice in the IF groups were allowed to be fed ad libitum for every other week. For each age group, one of them was set as the IF group and the other, the AL (sham) group. Water was available ad libitum for all mice, regardless of group allocation. The recording operations were performed 8 weeks later when the mice were put on fasting, and filming was between 8:00–10:00 am. Both the IF and AL group animals were fasted overnight with no access to food for 8–10 h before recording. The length of each video was 40 min. Details of Dataset C are shown in Table 6.

### Data processing

Like most conventional multiple animal tracking approaches, we divided the frames into occlusion frames and non-occlusion frames as part of preprocessing. The first step was segmentation [45, 46], where given a frame of video, it was necessary to distinguish between pixels associated with subjects (i.e., the mice) or the background. In this step, each frame was normalized with respect to its average intensity to correct for illumination fluctuations. It was also possible to implement background subtraction by generating a model of the background calculated as the average of a collection of frames obtained via subsampling the video. And according to the standard notation in the terminology the image processing field, here we refer to a collection of connected pixels that are not part of the background as a *blob*. The second step was frame classification, where frames were divided into occlusion frames and non-occlusion frames. In the open field test, the number of rodent subjects was a constant value declared by the user. It was possible to perform a comparison between the number of calculated experimental subjects and the number of actual experimental subjects to distinguish whether or not frame occlusion occurred. Put simply, when the number of blobs in a frame corresponded to the actual number of subjects, we considered this frame as a non-occlusion frame. In contrast, if the number of blobs and subjects did not match, we designated this an occlusion frame.

### Tracking in non-occlusion frames

In non-occlusion frames, the mice are not occluded by default. Thus, one blob can be used to represent one individual. In this case, blob data can be used to generate continuous individual trajectories that track the motion of individual subjects. In videos with high frame rates, a rodent’s location in space does not change too much in the gap between two adjacent frames. Therefore, if we overlay two adjacent frames into one image, the pair of blobs representing the same individual would share a large number of pixels in space. As such, in adjacent frames, the blob with the most overlapping pixels inherits the identification of the blob in the previous frame and the identifications can be assigned for every blob frame-by-frame. Technologies in non-occlusion tracking are simple and validated [47].

### Tracking in occlusion frames

Because of the occlusion of subjects, some blobs in occlusion fragments would contain multiple individuals in space. Therefore, we cannot assign identification directly like the operation in non-occlusion frames. In this study, the tracking operation in occlusion frames consisted of three main steps. The first step was ear labeling, and the ear was used for tracking in occlusion frames as opposed to the whole body of subjects. Following this, the object detection network was used to extract the location of ears in occlusion frames. The last step was using a dual-Siamese network to assign identification to located ears.

### Ear labeling

Since the video in Dataset A only contained a single rodent individual, the labeling could be completed by implementing two embedded single-target tracking operations with manual calibration. The first embedded operation was used to track the entire body of the subject in order to build a new video with cropped frames (the frames only contained the region of the rodent’s body). The second one was used to extract ears in the video for labeling in the original video clip. Due to the featureless background of the original video, the single target tracking operations could be simply replaced by two threshold segmentation processing. The first one was used to segment rodents and the background. The second one was for the ears and the body.

### Ear detection network

**Fig. 6:**
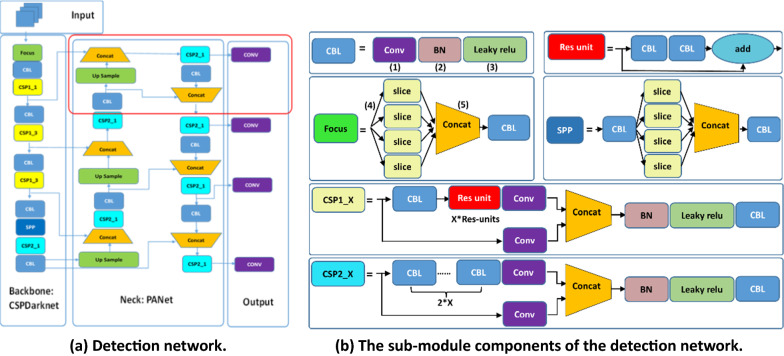
The structure of the proposed object detection network. **a** The detection network consists of three main parts: The (1) Backbone, a replaceable convolutional neural network for clustering and forming image features from fine and coarse gained images; The (2) Neck, a series of network layers for fusing and combining image features which are then sent to the predicting network, and (3), The Output, a network for prediction of image features, generation of bounding boxes and prediction of results. **b** The sub-module components of the detection network: (1) Convolutional layer. (2) Batch normalization operation. (3) Leaky Relu activation function. (4) Slicing operation. (5) Concatenate function puts slices into a block with 4X channels

Because of the extremely small size of mouse ears, the conventional detection network lacked interpretability of extremely small size objects, resulting in a low accuracy. In this case, improving the detection of small objects was necessary. In this step, we applied YOLOv5 [28] as the prototype framework due to its flexibility in modification to improve it (Fig. 6a).

As shown in Fig. 6, the EDDSN consists of three main parts: the Backbone, the Neck and the Output (the Head). The Backbone module is a convolutional neural network that aggregates and forms image features at different granularities. The Neck module is a series of layers to mix and combine image features which are passed forward to prediction.Then, the features from the Neck module were input into the Output module that used convolution layers to achieve ear detection. The sub-module components of the EDDSN are shown in more detail in Fig. 6b. The Focus module transferred spatial information to the channel dimension on the input images to help reduce the parameters used in the network to get faster inference without mAP penalty. The CBL module consisted of a Conv + BN + Leakyrelu activation function. The Conv is convolutional network and BN is batch normalized processing. The Res-unit, which is borrowed from the residual structure of the Resnet network, is used to build a deeper network. The CSP1_n is borrowed from the CSPNet network structure, is composed of a CBL module, a Res-unit module, and Conv and Concate. The CSP2_n is borrowed from the CSPNet network structure, which consists of Conv and n Res-unit modules. The Concate module is the Focus structure, which first concatenates multiple slice results, and then feeds them into CBL module; the SSP module uses the maximum pooling method to perform multi-scale fusion [48, 49] The images were first input to the Backbone for feature extraction, and then fed to PANet for feature fusion. Finally, the Head is the output of the detection results.

Similar to other methods in the same field, transfer learning methods using pre-trained models can shorten the training time and improve accuracy. Here, we tested the performance of the YOLOv5 models with and without pre-trained weights, and the results are shown in Table 7. It can be seen that on *mAP at 0.5* and *mAP at 0.5:0.9* (mean average precision at *IoU* is 0.5 and from 0.5 to 0.9), the performance of the models with pre-trained weights performs relatively better.

**Table 7:** Performance difference between pretrained and non-pretrained models

Model (Large CSPDarknet as backbone)	mAP at 0.5 %	mAP at 0.5:0.9 %
Improved framework with pretrained	**97.07** **(P=0.63)**	**64.32** **(P=0.0017)**
Original YOLOv5 with pretrained	96.10	58.58
Improved framework without pretrained	96.82	59.73
Original YOLOv5 without pretrained	96.18	58.15

Due to the requirement to use transfer learning strategy in this study, the pretrained weight was loaded to model Backbone (CSPDarknet) for improving performance and this part of the framework cannot be modified in structure (illustrated by the box marked as Backbone: CSPDarknet in Fig. 6a). The Model Neck is an inverted pyramid structure similar to PANet. And in this case, because of the inability to make modifications in the backbone part, the improvement could only be implemented on the Neck and Head. Instead of the original structure, the improved Neck and Head have the fourth connection of information stream from the low-level layer of the model’s Backbone (illustrated as the red box in Fig. 6a). This modification would improve the pyramid structure for better performance in obtaining low-level information and detailed information, thereby making a better performance for detecting objects of extremely small size.

### Identification assignment based on a dual Siamese network

**Fig. 7:**
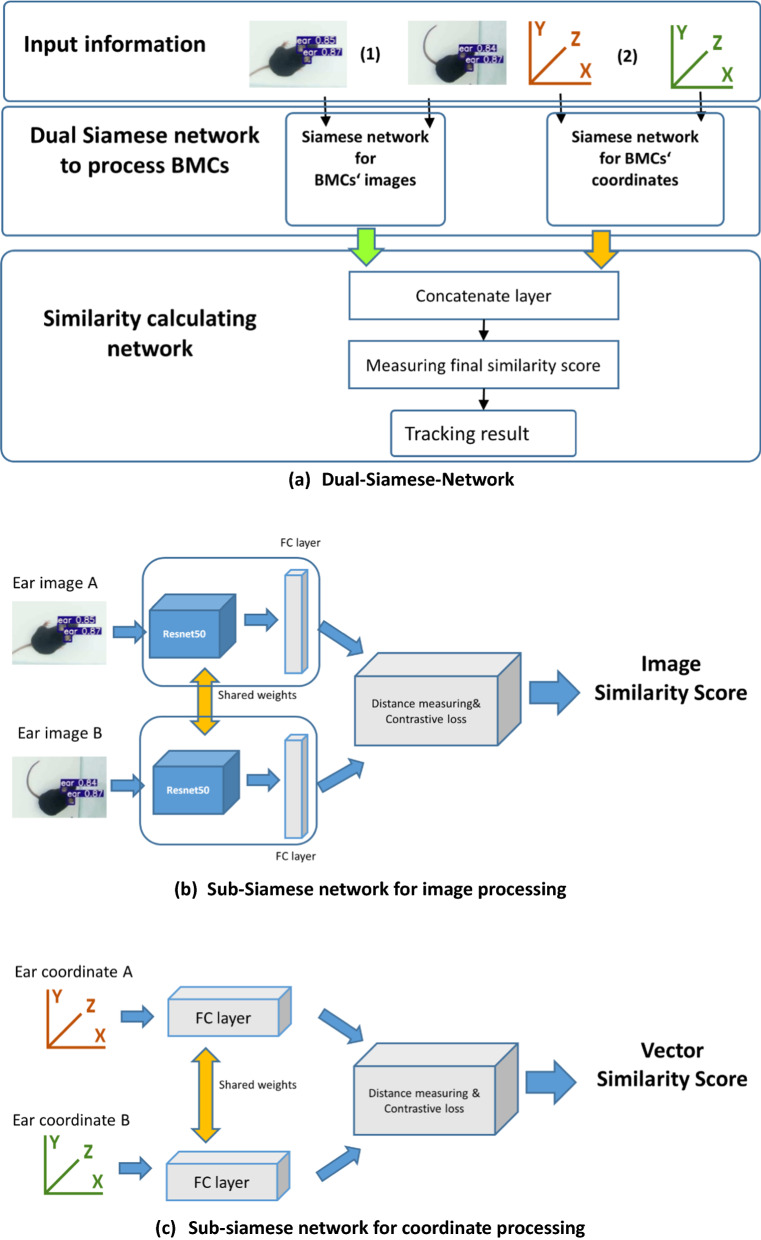
The structure of the proposed tracking network. **a** Dual-Siamese-Network: Input information: (1) images as input 1; (2) spatial information as input 2; Siamese network to process ears: the two networks in each Siamese-network are identical, with shared weight matrices at each layer; Similarity calculating network: using outputs of Siamese network to calculate the similarity of ears in adjacent frames to assign identifications; **b** Sub-siamese network for image processing; **c** Sub-siamese network for coordinate processing

Essentially, multiple object tracking in the video stream is a kind of identification assignment in adjacent frames. For ear tracking, it was necessary to associate each cropped image of ears in a frame with the ones in the previous frame. As we know, the biometrics characteristic extracted from a frame of a high-speed video has the uniqueness of morphology with the ones of the same individual in adjacent frames, as well as spatial information. Therefore, the similarities of image characteristics and spatial information of ears can be used as measurement metrics to implement identification assignment. Hence, we propose a fusion framework with dual Siamese-networks as Backbones that can process both spatial information and image feature information. The network structure is shown in Fig. 7. The spatial information and image feature information of a single ear in two adjacent frames are processed respectively by two independent Siamese networks [50–52]. Since the inputs of two independent Sub-Siamese-Networks are not of the same kind, the architectures of each are different. The Siamese network for processing images (as shown in Fig. 6b) is like another traditional Siamese network for matching images, in that it needs a convolutional network to extract features. Therefore, ResNet50 [50] was selected to perform this function. However, in the sub-network for processing coordinates, this convolutional architecture was omitted since the coordinate is input as a vector. The network parts described share the weights during training, so that the paired data pass through the exact same network architecture. Then, both sub-networks feed the vectors into the similarity checker with the contrastive loss [51, 52] to measure the similarity scores between image pairs and coordinate pairs. Finally, the results are concatenated as the input for another full connected network to finally obtain the similarity measurement to complete identification assignment.

### Generation of the final tracking results

Generally, the final tracking result is a combination of the results in occlusion frames and non-occlusion frames. The key to the combination is to link the tracking trajectories in both kinds of frames. Here, we used a frame-classifying operation to make every occlusion fragment incorporate one previous frame and one subsequent frame (these frames were non-occlusion frames). And then, these frames were employed for tracking using both strategies (the one for occlusion and the one for non-occlusion frames) and were assigned with the same identification to link the trajectories in two kinds of fragments.

### Experiments

#### Implementation details

##### Implementation details for ear detection network

The improved EDN was trained and tested on Dataset A. The clip for training and testing of ODN with 1080P original resolution and 60FPS frame rate, was used to take one image every other 5 frames. In total, 10314 frames were extracted randomly, which means that 20,628 images of mouse ears were used as the training input. And 2166 frames (4332 images) were used for testing. Due to the application of transfer learning strategy, the CSPdarknet [48] was used as the default Backbone model of EDN. In the training procedure, the resolution of the input video was 1280 × 720 and the number of epochs was 50, the batch size was set to 8 and the learning rate was set to 0.01. The main hyper-parameters of ear detection network are shown in Table 8.

**Table 8:** The main hyper-parameters of the ear detection network

Hyperparameters	The optimal setting
Input resolution	1280 × 720
Train epoch	50
Batch size	8
Optimizer	SGD
Initial learning rate	0.01
Final OneCycleLR learning rate	0.2
SGD momentum	0.937

There are 4 different pre-trained CSPdarknet models on MS-COCO [53] dataset ranging from the smallest one with 7.5 million parameters and 140 layers to the largest with 89 million parameters and 284 layers. In Table 9, which shows the ear detection performance of these 4 pre-trained models, we see that the “Large” pre-trained model achieves the highest *mAP at 0.5:0.9* (mean average precision from 0.5 to 0.9 interaction over union), thus, in this study, we used the “Large” pretrained model.

**Table 9:** Performance comparison of different volumes

Model volume	Layers	Parameter	*mAP at 0.5* %	*mAP at 0.5:0.9* %
Small	280	7.9 million	97.25	62.87
Medium	378	23.5 million	96.63	63.24
Large	476	51.7 million	97.07	64.32
Extreme	574	96.5 million	94.73	50.56

##### Implementation details for training and testing with dual-Siamese network

Since Dataset A is currently the only accessible dataset to do automatic labeling, this dual-Siamese-network was trained with the ear images and spatial information extracted from Dataset A. To be compatible with a lower frame rate video (the video in Dataset A has a high frame rate), the ear data used to train the target detection network was used here (i.e., one frame was taken every 3 frames, so the actual frame rate in training was only 20 frames per second). The images and spatial information in adjacent frames were automatically marked as positive samples, and the two with a time interval of more than one minute were automatically marked as negative samples. Obviously, the number of positive samples constructed in this way is limited, at most 24958. Negative samples can greatly exceed this amount. Here, we randomly selected 20,000 positive samples and 20,000 negative samples from it as the training dataset for the dual-Siamese network.

Clips in Dataset B for comparison of tracking system performance were used as input with original parameters and resolutions. Details are shown in Table 9.

Special attention should be paid to the video B6. The total number of frames in this video is 76191 by preprocessing of video-to-frames. But there was a fragment of human interference in the video. Therefore, the interfered with fragment needed to be removed (with 2108 frames in total) for the EDDSN-MRT to work properly. This left two non-interfered fragments (with 37,483 and 36,603 frames) which were processed using our methods.

##### Implementation details for rodent experiment validation on intermittent fasting intervention

Intermittent fasting (IF) is an increasingly popular dietary approach used for weight management and maintenance of overall health [54]. Tracking individual subject’s trajectories provides a noninvasive approach for the assessment of locomotion changes in animal models with different interventions. We collected data from 32 mice (n_18m-IF_ = 8, n_18m-AL_ = 8, n_3m-IF_ = 8, n_3m-AL_ = 8; Table 6) in Dataset C with our tracking system and subjected them to distributions of temporal features (e.g., velocity) analyses. Clips in Dataset C were used with original parameters and resolutions. By only evaluating spontaneous movement without any induced conditions, we demonstrated the usability and unbiased character of our framework for individual and social behavior monitoring in animal models. By applying the tracking system in this experiment, differences in group average and individual velocities and location distribution between the IF and AL groups can be observed.

### Methods for comparison

#### Ear detection methods

To show the effectiveness of the proposed ear detection network, we compared it with several object detection methods as follows:

1. YOLOv3

YOLOv3 is the 3^rd^ version of YOLO series [48]. It employs a multi-scale schema, predicting bounding boxes at different scales. This allows Yolov3 to be more effective for detecting smaller targets when compared to the previous version YOLOs. It uses dimension clusters as anchor boxes in order to predict bounding boxes around the desired objects in given images. Logistic regression is used to predict the object score for a given bounding box.

Here, it was trained with Adam optimizer with a learning rate of 0.001, the number of epochs set to 50, batch size set to 8, resolution at 1280 × 736 (YOLOv3 network only accepts resolutions whose value is an integer multiple of 32), and momentum at 0.9.

2. YOLOv5

The YOLOv5 model is a detector consisting of a cross-stage partial network (CSPNet, as shown as Fig. 5b) [26] backbone, and a “Head” model with Path Aggregation Network (PANet) for instance segmentation. The Backbone network combined with a Spatial Pyramid Pooling (SPP) network [56] that was used to resist object deformation. The model was trained with SGD optimizer with a learning rate of 0.01, epoch set to 50, batchsize set to 8, resolution at 1280 × 720, and momentum at 0.937.

3. EfficientDet

The EfficientDet is an object detection framework built by the Google Brain team [27]. It achieved state-of-the-art accuracy on the popular MS-COCO dataset [53]. It includes pre-trained models classed from D0 to D7, which each have different numbers of parameters (D0 with the fewest and D7 with the highest). In the application purpose considered (for video frame processing, there is the requirement of execution speed), the EfficientDet D1 was selected. It was trained with SGS optimizer with a learning rate of 0.00005, epoch set to 50, batchsize set to 8, and momentum set to 0.9.

### Animal tracking methods

To show the performance of the proposed EDDSN-MRT, we compared it with several existing state-of-the-art animal tracking methods as follows:

1. Toxtrac

Toxtrac [25] is an automated open-source executable software for image-based tracking that can simultaneously handle several subjects for monitoring in laboratory environments. It can be used for high-speed tracking of insects, fish, rodents or other species to provide useful locomotor information in animal behavior experiments. It was implemented with the threshold set to 90, minimum Object size set to 2000, maximum Object size set to 40,000, and maximum Distance/Frame set to 100. The numbers of individuals corresponded to the number of mice in the video.

2. idtracker.ai

Idtracker.ai [24] is an image-based multi-animal tracking system that uses convolutional neural networks to identify each of the individuals in the video. It uses offline training strategy. In the videos with a higher density of individuals, idtracker.ai extracts frames of the single individuals to train an image classification network to identify individuals. It was implemented with the area set as [2000,4000], and intensity was set as 80. The number of blobs was set equal to the number of individuals featured in each video. The range was set equal to the number of frames of each video.

### Evaluation metrics

#### Metrics for ear detection

As the methods for many conventional object detection networks, the mean average precision– *mAP at 0.5* and *mAP at 0.5:0.9* are introduced as evaluation metrics to quantitatively measure the detecting performance. These evaluation metrics are based on the Intersection over Union (IOU) of the ground truth and detected bounding boxes.

$$IoU = \frac{Area of overlap between bounding boxes}{{Area of union between bounding boxes}}$$

We set the threshold to determine whether the object is a true positive. *mAP at 0.5* means when *IoU* is set to 0.5, the average precision of all categories is calculated independently and then averaged by the number of categories. In addition, *mAP at 0.5:0.9* illustrates the average *mAP* over different *IoU* thresholds (from 0.5 to 0.9, in steps of 0.1).

#### Metrics for multiple rodent tracking

We used the widely accepted metric multi-object tracker accuracy (MOTA) proposed in the 2016 MOT Challenge [61]. To evaluate the performance of trackers, we used the py-motmetrics library. The MOTA tracking performance measure used in this study is the most commonly used metric to benchmark MOT solutions (Eq. 3).

3 $$MOTA = 1 - \frac{{\mathop \sum \nolimits_{t} FN_{t} + FP_{t} + IDSW_{t} }}{{\mathop \sum \nolimits_{t} GT_{t} }}$$

where false negative (FN), false positive (FP) and identity switch (IDSW) are the three types of errors that occur. False negatives are defined as an object that is not tracked, false positives are defined as tracked objects which should not be tracked, and identity switches describe two objects that should be tracked but they swap identities. The GT indicates the absolute number of individual identities. The direct output of the tracker is a series of IDs, which are mapped to our manually annotated tracks. The result of this implementation is a large number of ‘‘tracklets’’ (partial tracks), subsets of which belong to individual identities.

This paper also introduces the metric ICR (ID Correct Rate). The ICR means the number of images correctly identified over the total number of individual images validated [24, 25] (Eq. 4).

4 $$ICR = 1 - \frac{{\mathop \sum \nolimits_{t} Miss_{t} + Switch_{t} + Drift_{t} }}{{\mathop \sum \nolimits_{t} GT_{t} }}$$

where the missing identities (Miss), the switched identities (Switch) and the drifted identities (Drift) are the three types of errors that occur. Via the mapping between output and manually annotated tracks, it can be identified when the tracker is not able to detect an object (missing identities), when the tracker detects an object with the wrong position (drifting identities), or when the identities (two or more) tracks are switched.

It must be emphasized that these methods are designed based on a constant number of experimental subjects. this design strategy would prevent the tracker from providing more false positive trajectories than the real number of experimental individuals..

### Statistical analysis

For the proportion indicators such as ICR, MOTA and mAP, we performed the "N-1" Chi-squared test to assess for significant effects. To determine whether there were significant differences between two variables, we first performed the Shapiro–Wilk test and Levene’s test to assess for normality and homogeneity of variance, respectively. Following, for normally distributed variables, we performed Student’s T test, and for non-normal variables we performed the Wilcoxon Rank Sum Test. Specifically, for testing the velocity of different groups, we used the average velocity of all individuals in a particular group in 8 time periods as a variable, and for testing the dwell distribution of different groups, we used the summed histogram values of all individuals of a group in all bins as a variable. For the two-dimensional standard deviation to measure the individual distribution of mice, because two-dimensional standard deviation is a scalar, it cannot be tested for significance. All statistical analyses were performed using MedClac, version 20.027, MedCalc Software Ltd, Belgium.

## Supplementary Information

**Additional file 1: **Video demonstrating the performance of EDDSN-MRT applied on the clip with two individuals.

**Additional file 2: ** Video demonstrating the performance of EDDSN-MRT applied on the clip with 4 individuals.

## Data Availability

Rodent public dataset (Dataset B) is freely-available and can be downloaded through (https://idtrackerai.readthedocs.io/en/latest/data.html). The unpublished datasets (Dataset A and C) that support the findings of this study are available from the Xiangya School of Medicine, Central South University, but restrictions apply to the availability of these data, which were used under license for the current study, and are therefore not publicly available. Dataset A and C are however available from the authors upon reasonable request and with permission of Xiangya School of Medicine, Central South University.

## References

[1] Tecott LH, Nestler EJ (2004). Neurobehavioral assessment in the information age. Nat Neurosci.

[2] Brunner D, Nestler E, Leahy E (2002). In need of high-throughput behavioral systems. Drug Discov Today.

[3] Houle D, Govindaraju DR, Omholt S (2010). Phenomics: the next challenge. Nat Rev Genet.

[4] Askenasy J-JM (2001). Approaching disturbed sleep in late Parkinson's disease: first step toward a proposal for a revised UPDRS. Parkinsonism Related Disord.

[5] Vogel-Ciernia Annie (2013). The neuron-specific chromatin regulatory subunit BAF53b is necessary for synaptic plasticity and memory. Nature Neurosci.

[6] Lewejohann Lars (2009). Behavioral phenotyping of a murine model of Alzheimer’s disease in a seminaturalistic environment using RFID tracking. Behavior Res Methods.

[7] Kalueff Allan V (2016). Neurobiology of rodent self-grooming and its value for translational neuroscience. Nature Rev Neurosci.

[8] Crawley JN (2007). Mouse behavioral assays relevant to the symptoms of autism. Brain Pathol.

[9] Moy SS (2004). Sociability and preference for social novelty in five inbred strains: an approach to assess autistic-like behavior in mice. Genes Brain Behav.

[10] Nadler JJ (2004). Automated apparatus for quantitation of social approach behaviors in mice. Genes Brain Behav.

[11] F Chaumont de, et al. "Live Mouse Tracker: real-time behavioral analysis of groups of mice. BioRxiv. 2018;345132.

[12] K Gregory, et al. Automated mouse behavior recognition using VGG features and LSTM networks. Proc Vis Observ Anal Vertebrate Insect Behav Workshop (VAIB). 2016.

[13] Jiang Zheheng (2021). Multi-View Mouse Social Behaviour Recognition With Deep Graphic Model. IEEE Trans Image Process.

[14] Sun ShiJie (2019). Deep affinity network for multiple object tracking. IEEE Trans Pattern Anal Mach Intell.

[15] Hou, Xinyu, Yi Wang, and Lap-Pui Chau. "Vehicle tracking using deep sort with low confidence track filtering." 2019 16th IEEE International Conference on Advanced Video and Signal Based Surveillance (AVSS). IEEE, 2019.

[16] Itskovits Eyal (2017). A multi-animal tracker for studying complex behaviors. BMC Biol.

[17] Rao SR (2019). Small animal video tracking for activity and path analysis using a novel open-source multi-platform application (AnimApp). Sci Reports.

[18] Geuther BQ (2019). Robust mouse tracking in complex environments using neural networks. Commun Biol.

[19] Yamanaka O, Takeuchi R (2018). UMATracker: an intuitive image-based tracking platform. J Exp Biol.

[20] Tang X, Sanford DL (2002). Telemetric recording of sleep and home cage activity in mice. Sleep.

[21] Johansson C, Thorén P (1997). The effects of triiodothyronine (T3) on heart rate, temperature and ECG measured with telemetry in freely moving mice. Acta Physiol Scand.

[22] Mills PA, Huetteman DA, Brockway BP (2000). A new method for measurement of blood pressure, heart rate, and activity in the mouse by radiotelemetry. J Appl Physiol.

[23] Dennis RL (2008). Appearance matters: artificial marking alters aggression and stress. Poult Sci.

[24] Romero-Ferrero Francisco (2019). Idtracker. ai: tracking all individuals in small or large collectives of unmarked animals. Nat Methods.

[25] Rodriguez Alvaro (2018). ToxTrac: a fast and robust software for tracking organisms. Methods Ecol Evol.

[26] C-Y Wang, et al. "CSPNet: a new backbone that can enhance learning capability of CNN." Proceedings of the IEEE/CVF conference on computer vision and pattern recognition workshops. 2020.

[27] T Mingxing, R Pang, V Quoc Le. "Efficientdet: Scalable and efficient object detection." Proceedings of the IEEE/CVF conference on computer vision and pattern recognition. 2020.

[28] J Glenn, et al. "ultralytics/yolov5" Zenodo. (2020).

[29] Idayu NF (2011). Antidepressant-like effect of mitragynine isolated from Mitragyna speciosa Korth in mice model of depression. Phytomedicine.

[30] Lee J-E (2020). Aging increases vulnerability to stress-induced depression via upregulation of NADPH oxidase in mice. Commun Biol.

[31] Fond G (2013). Fasting in mood disorders: neurobiology and effectiveness. a review of the literature. Psychiatry Res.

[32] Zhang Kai (2016). P2X7 as a new target for chrysophanol to treat lipopolysaccharide-induced depression in mice. Neurosci Lett.

[33] Sulakhiya Kunjbihari (2016). Lipopolysaccharide induced anxiety-and depressive-like behaviour in mice are prevented by chronic pre-treatment of esculetin. Neurosci lett.

[34] Hussin NM (2013). Efficacy of fasting and calorie restriction (FCR) on mood and depression among ageing men. J Nutr Health Aging.

[35] Michalsen Andreas (2009). Hunger and mood during extended fasting are dependent on the GNB3 C825T polymorphism. Ann Nutr Metab.

[36] Teng Nur Islami, Fahmi Mohd (2011). Efficacy of fasting calorie restriction on quality of life among aging men. Physiol Behavior.

[37] Swoap Steven J (2006). The full expression of fasting-induced torpor requires β3-adrenergic receptor signaling. J Neurosci.

[38] Kanizsai P (2009). Energetics of fasting heterothermia in TRPV1-KO and wild type mice. Physiol Behav.

[39] JW Hudson. Shallow daily torpor: a thermoregulatory adaptation. Strategies in cold: Natural torpidity and thermogenesis. 1978.

[40] Webb Jagot GPSA, Jakobson ME (1982). "Fasting-induced torpor in Mus musculus and its implications in the use of murine models for human obesity studies." comparative biochemistry and physiology. Comparat Physiol.

[41] Webb GP (1980). Effects of fasting on thermoregulation in normal and obese mice. IRCS Med Sci Biochem.

[42] Brown Jason CL, James FS (2010). Mitochondrial metabolism during fasting-induced daily torpor in mice. Biochimica et Biophysica Acta Bioenerg.

[43] Swoap SJ, Weinshenker D (2008). Norepinephrine controls both torpor initiation and emergence via distinct mechanisms in the mouse. PLoS ONE.

[44] Morton SR (1978). Torpor and nest-sharing in free-living Sminthopsis crassicaudata (Marsupialia) and Mus musculus (Rodentia). J Mammal.

[45] Pal NR, Pal SK (1989). Object-background segmentation using new definitions of entropy. IEE Proc E-Comput Digital Tech.

[46] Kim K (2005). Real-time foreground–background segmentation using codebook model. Real-Time Imaging.

[47] Chen J-T et al. 2001 "Boundary element analysis for the Helmholtz eigenvalue problems with a multiply connected domain" Proceedings of the Royal Society of London. Series Mathematical, Physical and Engineering Sciences. 457(2):2521–2546.

[48] R Joseph, A Farhadi. "Yolov3: An incremental improvement." arXiv preprint arXiv:1804.02767. (2018).

[49] Liu, Shu, et al. "Path aggregation network for instance segmentation." Proceedings of the IEEE conference on computer vision and pattern recognition. 2018.

[50] H Kaiming, X Zhang, S Ren. "J. Sun, J. Deep residual learning for image recognition." Proceedings of the IEEE conference on Computer Vision and Pattern Recognition. 2015.

[51] Payer Christian, Frangi Alejandro F, Schnabel Julia A, Davatzikos Christos, Alberola-López Carlos, Fichtinger Gabor (2018). "Instance segmentation and tracking with cosine embeddings and recurrent hourglass networks. International conference on medical image computing and computer-assisted intervention.

[52] Liu Lihao (2019). Multi-task deep model with margin ranking loss for lung nodule analysis. IEEE Trans Med Imaging.

[53] Lin T-Y, et al. 2014 Microsoft coco Common objects in context. David Fleet, Tomas Pajdla, Bernt Schiele, Tinne Tuytelaars (Eds). European conference on computer vision. Cham: Springer

[54] Mattson MP, Longo VD, Harvie M (2017). Impact of intermittent fasting on health and disease processes. Ageing Res Rev.

[55] Andrea Di F (2018). A time to fast. Science.

[56] Mattison Julie A (2017). Caloric restriction improves health and survival of rhesus monkeys. Nat Commun.

[57] Meynet O, Ricci J-E (2014). Caloric restriction and cancer: molecular mechanisms and clinical implications. Trends Mol Med.

[58] Nencioni Alessio (2018). Fasting and cancer: molecular mechanisms and clinical application. Nat Rev Cancer.

[59] Speakman JR, Mitchell SE (2011). Caloric restriction. Mol Aspects Med.

[60] He Kaiming (2015). Spatial pyramid pooling in deep convolutional networks for visual recognition. IEEE Trans Pattern Anal Mach Intell.

[61] M Anton, et al. "MOT16: a benchmark for multi-object tracking." arXiv preprint arXiv:1603.00831. 2016.

